# A Rapid and Marked Response to Guselkumab in Extensive Plaque Psoriasis: A Case Report

**DOI:** 10.7759/cureus.107869

**Published:** 2026-04-28

**Authors:** Eduardo Oropesa, Yulio L Aguila, Berenice D Pena Reina, Isnel D Vargas Batista, Yisenia Pereda Gonzalez, Maria D Milanes Chongon

**Affiliations:** 1 Dermatology, Advanced Dermatology and Cosmetic Surgery, Lehigh Acres, USA; 2 Primary Care, Adtremed Research Clinic, Land O Lakes, USA; 3 Clinical Research Department, Primary Care - Research Initiative (PCRI), Miami, USA; 4 Research, Deluxe Health Center, Tampa, USA; 5 Cardiology, Hermanos Ameijeiras Hospital, Havana, CUB; 6 Primary Care, Family Health Centers, Louisville, USA

**Keywords:** biologic therapies, body surface area, guselkumab, plaque psoriasis, "psoriasis, psoriasis area severity index (pasi)

## Abstract

Psoriasis is a chronic immune-mediated inflammatory skin disease associated with significant morbidity and impaired quality of life. The introduction of biologic therapies, particularly interleukin (IL)-23 inhibitors such as guselkumab, has significantly improved outcomes in patients with moderate-to-severe disease, achieving high rates of skin clearance. However, clinical response is typically gradual, with maximal improvement occurring after 12-16 weeks, and rapid, near-complete clearance following a single dose remains uncommon and not well described.

We present the case of a 58-year-old female with severe plaque psoriasis involving approximately 90% of body surface area (BSA), associated with intense pruritus and pain due to skin breakdown. Prior treatments had failed to achieve adequate disease control. Guselkumab was initiated according to standard dosing protocols. At the four-week follow-up after a single injection, the patient demonstrated a marked clinical response, with a reduction in BSA involvement from 90% to 5%, along with significant improvement in erythema, scaling, and plaque thickness, and substantial symptomatic relief. No adverse effects were observed.

This report highlights an unusually rapid and pronounced response to guselkumab. However, given the single-case design, this observation should be interpreted with caution and may represent a hypothesis-generating finding that underscores interindividual variability in treatment response. Further studies are needed to better understand predictors of rapid response and their implications for personalized management of psoriasis.

## Introduction

Psoriasis is a chronic, immune-mediated inflammatory skin disease characterized by the formation of well-demarcated, erythematous, and scaly plaques on various parts of the body, most commonly the scalp, elbows, knees, and trunk. In some patients, it affects only small areas, while in others it becomes more widespread [1]. It is estimated to affect between 2% and 3% of the global population, with a higher incidence in Nordic countries and a lower incidence in equatorial regions [2]. It most frequently affects individuals between the ages of 15 and 35 years [3].

The burden of the disease extends beyond visible skin manifestations and significantly impacts patients’ quality of life, including psychosocial well-being [4]. The pathogenesis of psoriasis involves interactions between genetic predisposition and environmental triggers, affecting not only the skin but also the joints, the cardiovascular and metabolic systems, and psychological well-being. Immune dysregulation involves both innate and adaptive immune responses, with the TNF-α-IL-23-Th17 axis playing a central role in the maintenance of inflammation [5].

Until the 1990s, therapeutic options were limited to topical regimens, phototherapy, and systemic agents such as methotrexate or fumaric acid esters, which were associated with limited efficacy and potential adverse effects. However, the advent of biologic therapies has significantly transformed the treatment landscape. Since the introduction of tumor necrosis factor inhibitors, multiple targeted therapies have emerged, including interleukin (IL)-17 and IL-23 inhibitors, which are associated with high rates of skin clearance in patients with psoriasis. Guselkumab is a human monoclonal antibody targeting IL-23 and is approved by the U.S. Food and Drug Administration for the treatment of moderate-to-severe plaque psoriasis [6].

Guselkumab has demonstrated robust efficacy in moderate-to-severe plaque psoriasis, with a significant proportion of patients achieving Psoriasis Area and Severity Index (PASI) responses in clinical trials. Maximal response is typically achieved after 12-16 weeks, with gradual improvement observed over time. However, rapid and near-complete clearance within weeks following a single dose is uncommon and not well characterized in the literature. We present a case of extensive plaque psoriasis with approximately 90% body surface area (BSA) involvement, demonstrating an unusually rapid and marked clinical response, with near-complete clearance following a single dose of guselkumab, highlighting significant variability in treatment response.

## Case presentation

A 58-year-old female presented to the clinic with a diffuse rash involving her entire body. She reported intense pruritus as well as pain in areas of skin breakdown. Her symptoms had progressively worsened, significantly impacting her quality of life. The patient had a long-standing history of psoriasis and had not previously received biologic therapy. She had no history of psoriatic arthritis or relevant metabolic comorbidities, including obesity, diabetes mellitus, or dyslipidemia. No other clinical factors contributing to disease severity were identified.

**Figure 1:**
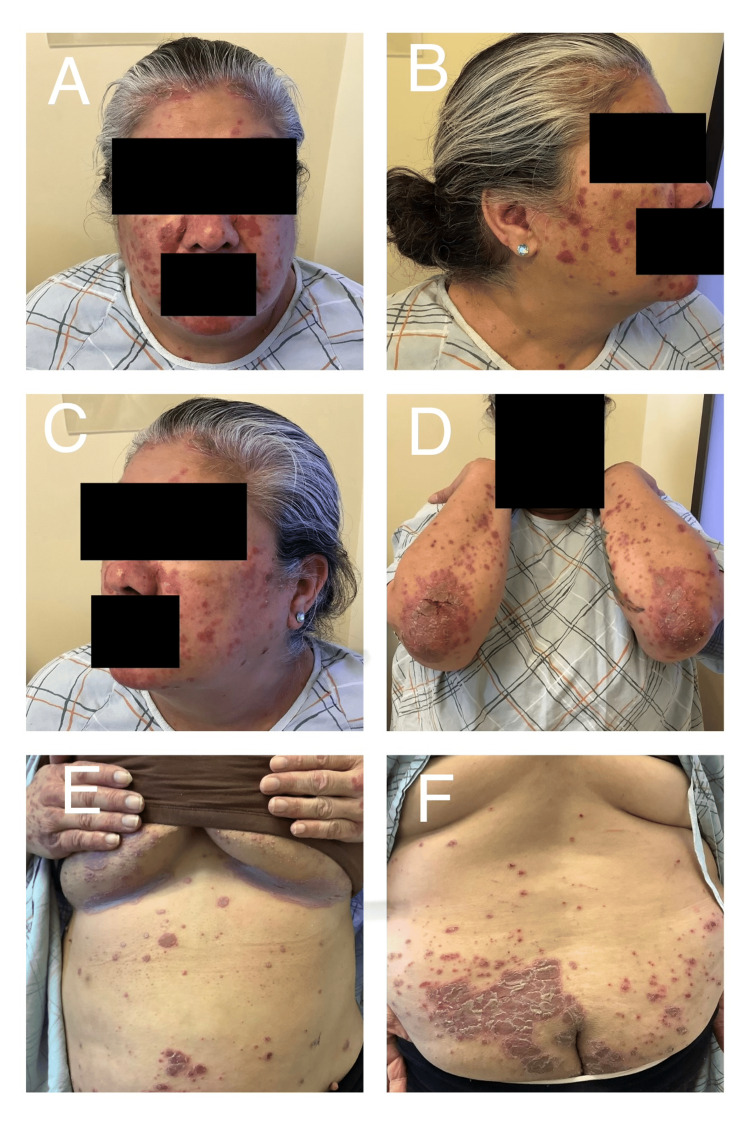
Baseline presentation of extensive plaque psoriasis prior to the initiation of guselkumab therapy (A) Frontal view of the face showing multiple erythematous, well-demarcated plaques with overlying scale, predominantly involving the cheeks, chin, and forehead. (B) Right lateral facial view demonstrating widespread erythematous papules and plaques with scaling. (C) Left lateral facial view with a similar distribution of inflammatory plaques affecting the cheek and perioral region. (D) Bilateral upper extremities showing numerous erythematous papules and plaques, with thick scaling and lichenification over the extensor surfaces, particularly at the elbows. (E) Anterior trunk view revealing scattered erythematous plaques and papules involving the inframammary region and abdomen. (F) Lower back and gluteal region demonstrating extensive, confluent erythematous plaques with thick adherent scale

On physical examination, there were widespread erythematous plaques with prominent scaling affecting the trunk, upper and lower extremities, and other anatomical regions. The extent of involvement was estimated at approximately 90% of the body surface area, consistent with severe plaque psoriasis. Areas of excoriation and skin disruption were noted, correlating with the patient’s reported pain and pruritus (Figure 1).

Erythrodermic psoriasis was considered in the differential diagnosis; however, the presence of well-demarcated plaques and the absence of systemic symptoms such as fever, chills, or hemodynamic instability supported a diagnosis of severe plaque psoriasis. Prior therapeutic interventions, including topical and systemic corticosteroids, topical calcineurin inhibitors, and vitamin D analogs, failed to achieve adequate disease control, prompting the initiation of biologic therapy. Standard pre-biologic screening, including evaluation for tuberculosis and viral hepatitis, was performed and found to be unremarkable.

**Figure 2:**
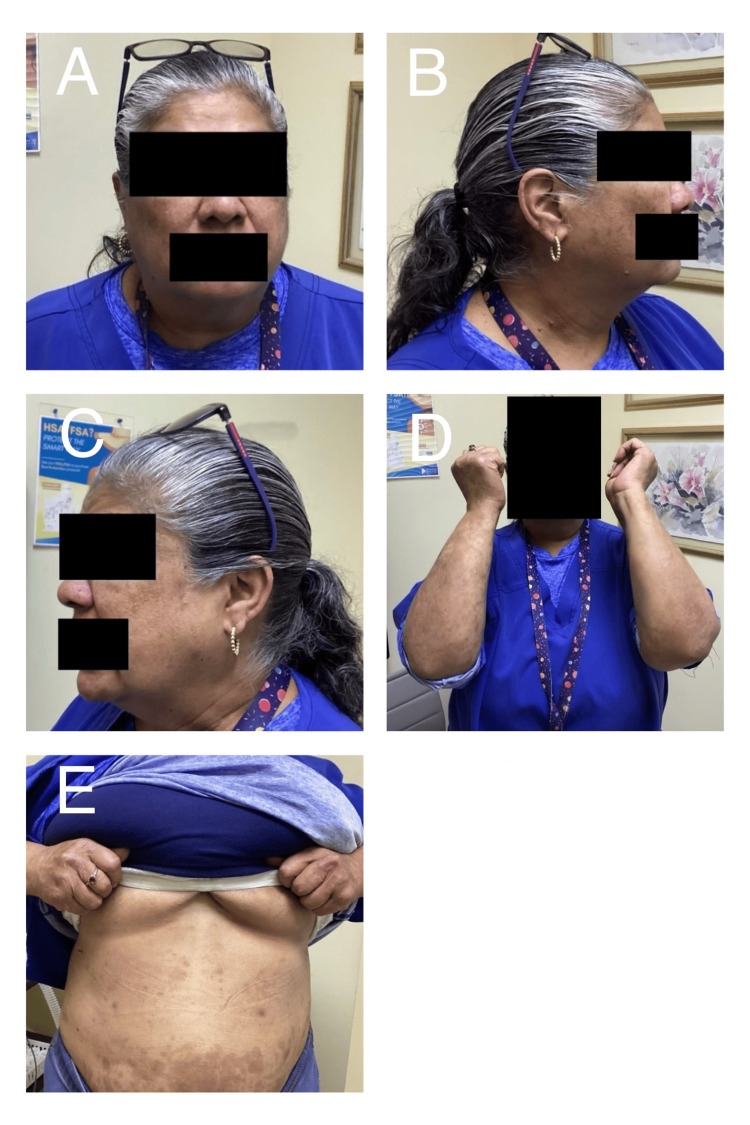
Near-complete clearance following a single dose of guselkumab (A) Frontal facial view demonstrating near-complete resolution of previously noted erythematous plaques, with minimal residual post-inflammatory changes and absence of significant scaling. (B) Right lateral facial view showing marked clearance of inflammatory lesions, with only faint residual erythema and post-inflammatory hyperpigmentation. (C) Left lateral facial view confirming substantial improvement, with resolution of active plaques and minimal residual discoloration. (D) Bilateral upper extremities demonstrating significant reduction in plaque burden, with resolution of thick scaling and only mild residual erythema. (E) Anterior trunk view showing near-complete clearance of prior lesions, with residual post-inflammatory hyperpigmentation and no active scaling or erythematous plaques

Treatment with guselkumab (100 mg subcutaneously) was initiated according to standard dosing protocols. Following administration of the first injection, the patient was monitored for clinical response and tolerability. At the four-week follow-up, before administration of the second dose, the patient demonstrated remarkable improvement in disease severity. The extent of psoriasis involvement decreased significantly, with BSA involvement decreasing from approximately 90% at baseline to 5%. In addition to the reduction in affected surface area, there was a notable improvement in erythema, scaling, and plaque thickness. The patient reported significant symptomatic relief, including decreased pruritus and improvement in pain, along with restoration of skin integrity in previously affected areas (Figure 2). No adverse effects or complications related to treatment were observed during the follow-up period.

## Discussion

The management of moderate-to-severe plaque psoriasis has evolved significantly with the introduction of biologic therapies, which provide targeted immunomodulation and improved clinical outcomes. These agents have demonstrated high efficacy in achieving substantial reductions in disease severity, with many patients reaching high levels of skin clearance. Among these therapies, guselkumab has demonstrated robust clinical efficacy, with a significant proportion of patients achieving PASI 90 and PASI 100 responses in clinical trials [7].

Despite these favorable outcomes, the majority of patients typically experience gradual improvement, with maximal clinical response occurring within approximately 12 to 16 weeks of therapy. While early responses to guselkumab have been reported in clinical trials, substantial improvement is generally progressive over time, with higher levels of clearance achieved at later time points. In pivotal clinical trials such as VOYAGE 1 and 2, early responses at week four are generally modest, with higher levels of clearance achieved at later time points [7,8]. The rapid and marked improvement observed in this case, with a reduction in BSA involvement from 90% to 5% within four weeks after a single injection, is therefore notable and suggests variability in treatment response. This degree of improvement over a short period highlights a response that is faster than typically expected based on available clinical data [9,10].

This accelerated clinical response may be explained by early interruption of key inflammatory pathways involved in psoriasis. The disease is driven by immune-mediated activation of T lymphocytes and downstream proinflammatory cytokines that sustain keratinocyte proliferation and chronic inflammation. Targeted inhibition of pathways mediated by interleukin-23 plays a central role in reducing inflammatory activity, and early suppression of these signals may result in rapid clinical improvement, particularly in patients with highly active inflammatory disease [7,10].

This case demonstrates a rapid and pronounced clinical response shortly after initiation of biologic therapy, emphasizing interindividual variability in treatment outcomes. Differences in immune pathway activation, disease chronicity, and prior treatment exposure may contribute to such variability. Patients with predominantly inflammatory lesions, as opposed to long-standing fibrotic plaques, may be more likely to exhibit rapid improvement following targeted therapy.

The clinical implications of this observation are significant. Early and substantial improvement may enhance patient adherence, improve quality of life, and provide reassurance regarding treatment efficacy. Additionally, recognition of variability in treatment response may help guide clinical expectations and support individualized treatment strategies in patients with severe psoriasis. These findings highlight the heterogeneity of treatment response in psoriasis and underscore the importance of individualized management approaches. While most patients follow a predictable trajectory of gradual improvement, this case illustrates that rapid and substantial responses can occur in selected individuals.

This report is limited by its single-case design, which limits generalizability and prevents drawing causal inferences. Additionally, the absence of long-term follow-up limits the assessment of sustained clinical response and the durability of the treatment effect. Despite these limitations, this case provides valuable clinical insights into variability in response to biologic therapy and emphasizes the potential for early, marked improvement in severe plaque psoriasis.

## Conclusions

This report demonstrates a rapid and marked clinical response to guselkumab in a patient with severe plaque psoriasis, with substantial reduction in BSA involvement within four weeks following a single dose, earlier than typically observed in clinical trials. This observation highlights the potential for variability in treatment response among patients with psoriasis and underscores that, in selected individuals, significant improvement may occur sooner than expected. Recognition of such variability may help inform clinical expectations and support individualized treatment approaches.
